# Climatic Variability Leads to Later Seasonal Flowering of Floridian Plants

**DOI:** 10.1371/journal.pone.0011500

**Published:** 2010-07-21

**Authors:** Betsy Von Holle, Yun Wei, David Nickerson

**Affiliations:** 1 Department of Biology, University of Central Florida, Orlando, Florida, United States of America; 2 Department of Statistics, University of Central Florida, Orlando, Florida, United States of America; Umea University, Sweden

## Abstract

Understanding species responses to global change will help predict shifts in species distributions as well as aid in conservation. Changes in the timing of seasonal activities of organisms over time may be the most responsive and easily observable indicator of environmental changes associated with global climate change. It is unknown how global climate change will affect species distributions and developmental events in subtropical ecosystems or if climate change will differentially favor nonnative species. Contrary to previously observed trends for earlier flowering onset of plant species with increasing spring temperatures from mid and higher latitudes, we document a trend for delayed seasonal flowering among plants in Florida. Additionally, there were few differences in reproductive responses by native and nonnative species to climatic changes. We argue that plants in Florida have different reproductive cues than those from more northern climates. With global change, minimum temperatures have become more variable within the temperate-subtropical zone that occurs across the peninsula and this variation is strongly associated with delayed flowering among Florida plants. Our data suggest that climate change varies by region and season and is not a simple case of species responding to consistently increasing temperatures across the region. Research on climate change impacts need to be extended outside of the heavily studied higher latitudes to include subtropical and tropical systems in order to properly understand the complexity of regional and seasonal differences of climate change on species responses.

## Introduction

During the past century, average annual global temperatures for land and ocean surfaces have increased at a rate near 0.6°C/century (1.1°F/century), however the trend has been three times larger since 1976, with some of the greatest increases in temperature occurring in the high latitudes [1]. Florida has been getting increasingly warmer, with the average annual temperature increasing by 0.02°C per decade, with temperatures in 2007 0.2°C warmer than in 1895 [2]. Spring events such as leaf unfolding and flowering are associated with changes in air temperature [3]. Changes in phenology (the timing of seasonal activities of animals and plants) over time may be the most responsive and easily observable indicator of environmental changes associated with global climate change [3], [4]. Global climate change has had pronounced effects on the developmental events of species; the majority of observed changes in phenology have occurred in the direction that would be expected under warming, occurring earlier in the season[5]. Climate change and species invasions are two of the biggest contributors to global change, yet their effects have typically been considered separately [6]. It is expected that most aspects of global climate change (e.g. increasing CO_2_, nitrogen deposition, etc.) will favor nonindigenous species because invasive species share traits that allow them to capitalize on these perturbations [7]. In fact, warming temperatures have allowed nonnative species to expand their ranges into areas where they previously could not survive and reproduce [6]. Florida has the second highest number of nonnative plant species in the US comprising 27% of the flora [8] and state, federal, and local agencies devoted approximately $250 million dollars for the control and eradication of invasive nonnative species in Florida from 1980 to 2007 [9]. Under conditions of climatic change, plants in Florida have the potential to expand or contract their ranges to areas where they are better suited to the environmental conditions. While it is not possible to conclusively separate range expansion of nonnative species from climate-induced range expansion, a response to climate change would be implicated by changes in the flowering phenologies of plant species corresponding to specific changes in climate.

We tested the hypothesis that global climate change has altered the reproductive phenologies of populations of high-impact nonnative plant species and their closely related native congeners, predicting that nonnative species will have greater responses to climatic change than natives. To investigate this, we focused on the change in reproductive status of 29 high impact invasive plant species and 41 closely related native species in Florida over historical time, using herbarium specimens (Supporting material available online, Table S1). Our first objective was to determine if temperature and precipitation levels have changed in Florida counties over time by season (spring, summer, fall, winter). For this, we analyzed historical climatic records for the entire record of climate data available for each county. Second, we determined if the flowering phenologies of highly invasive nonnative plant species as well as closely related native species have changed over time, using flowering records from herbarium specimens. We also evaluated the environmental drivers that affected changes in reproductive phenologies with statistical models that matched individual flowering times with climatic variables for all biogeographic regions of Florida.

## Methods

Twenty-nine species were chosen from the 133 Category I and II Florida Exotic Pest Plant Council (EPCC) species for their distinct, short reproductive phenologies (as in Primack et al. 2004) and because they occur in more than one region of Florida. Category I species are high impact invaders that alter native plant communities by displacing native species, change ecological functions or community structures, or hybridize with natives, based on documented ecological damage [10]. Category II species are invasive exotics that have increased in abundance or frequency but have not yet altered Florida plant communities to the extent shown by Category I species (FLEPPC 2007). These species may be ranked Category I, if ecological damage is demonstrated. We identified 41 native species which were the most closely related to these 29 nonnative species below the family level to compare climate change impacts and to compare differences between the flowering times of native and nonnative species with climate change [11]. We chose taxonomically related native species commonly found in Florida that had distinct, short reproductive phenologies in order to compare the nonnative species with their most similar native species. If there were no native congeners to the focal nonnative species, we chose the native species in the most taxonomically similar genera to the focal genus, below the family level [11], [12]. There were five nonnative species (*Casuarina cunninghamiana*, C. *equisitifolia*, C. *glauca*, *Eleagnus pungens*, and *Melia azeradach*) which were used in the analysis but which had no confamilial native species in Florida.

We recorded 6,218 herbarium specimens for the 70 study species which were collected from 1819 to 2008, the majority of the accessions collected between 1929 and 2007. We used 5,019 of these specimens for this analysis, less than that collected, either because there were no matching climatic data for that county at the time of flowering or the specimen was not flowering.

### Flowering data

We monitored the length of time that each of these species was historically in flower and fruit by using specimens from six herbaria in Florida (Archbold Biological Station, Fairchild Tropical Botanical Garden, Florida State University, University of Central Florida, University of Florida, and the University of South Florida). We utilized accessions from these herbaria to determine the date and season that these species flowered over historical time. Using online or physical herbarium specimens, we recorded reproductive status (budding, flowering, or fruiting), the county, and date of collection of each specimen [13]. The use of herbarium specimens has been demonstrated to be successful in documenting phenological responses to global change and have been found to be comparable to field observations [14].

### Climate data

Monthly climate data (minimum temperature, maximum temperature, precipitation) were obtained from each of the 57 Florida counties with weather stations. These weather stations were established as early as 1900 (12 counties) to as late as 1973, with the majority installed by 1960 (Florida Climate Center, Center for Ocean-Atmospheric Prediction Studies). Climate data were obtained through July 2007. If there were more than one weather station per county, we averaged the monthly climate data for those stations for each climate variable.

### Statistical Analyses

#### Climatic trends by county

We performed regression analyses of monthly temperature and precipitation data for 57 Florida counties to determine if these climatic factors have changed over historical time. We used the entire record of climate data collection for each county, which ranged from 35 to 108 years (Table S2). To ascertain if the stations with more recent data would capture greater warming trends than the stations which covered longer time periods, we ran a Fishers Exact Test. In this test, significance (significantly positive, significantly negative, nonsignificant) and year category (35–49, 50–99, 100+ years) were the variables.

Low temperatures in the winter, high temperatures in the summer, high and low temperature extremes, as well as water availability are among the factors that limit plant distributions [15]. Thus, we analyzed the average minimum winter (December, January, February) and spring (March, April, May) temperatures, the average maximum summer (June, July, August) and fall (September, October, November) temperatures and mean precipitation for each county and season over time to understand how these climatic variables, which may influence plant reproduction, vary in Florida by season and county. To determine if autocorrelation occurred between years, we tested the residuals from the ordinary least squares regression for serial correlation using the generalized Durbin-Watson statistic [16]. If autocorrelation was detected, autoregressive error terms of the appropriate order were added to the model until the generalized Durbin-Watson statistic indicated there was no autocorrelation. Then the model was refitted, using the method of maximum likelihood with the autoregressive error terms of the required order.

#### Patterns of flowering time

To determine shifts in flowering time for each species, we performed t-tests on the difference between average flowering dates that occurred in an earlier time period (1890–1969) to those collected from 1970 to 2008. Only those species with more than ten specimens in both the earlier and later time periods were used for the analyses. Owing to low sample sizes when the data were separated by species, biogeographic region and the two time periods, we summed the data for each species across all biogeographic regions of the state. If the data were not normally distributed, we performed Mann-Whitney tests.

#### Biogeographic regions

**Figure 1:**
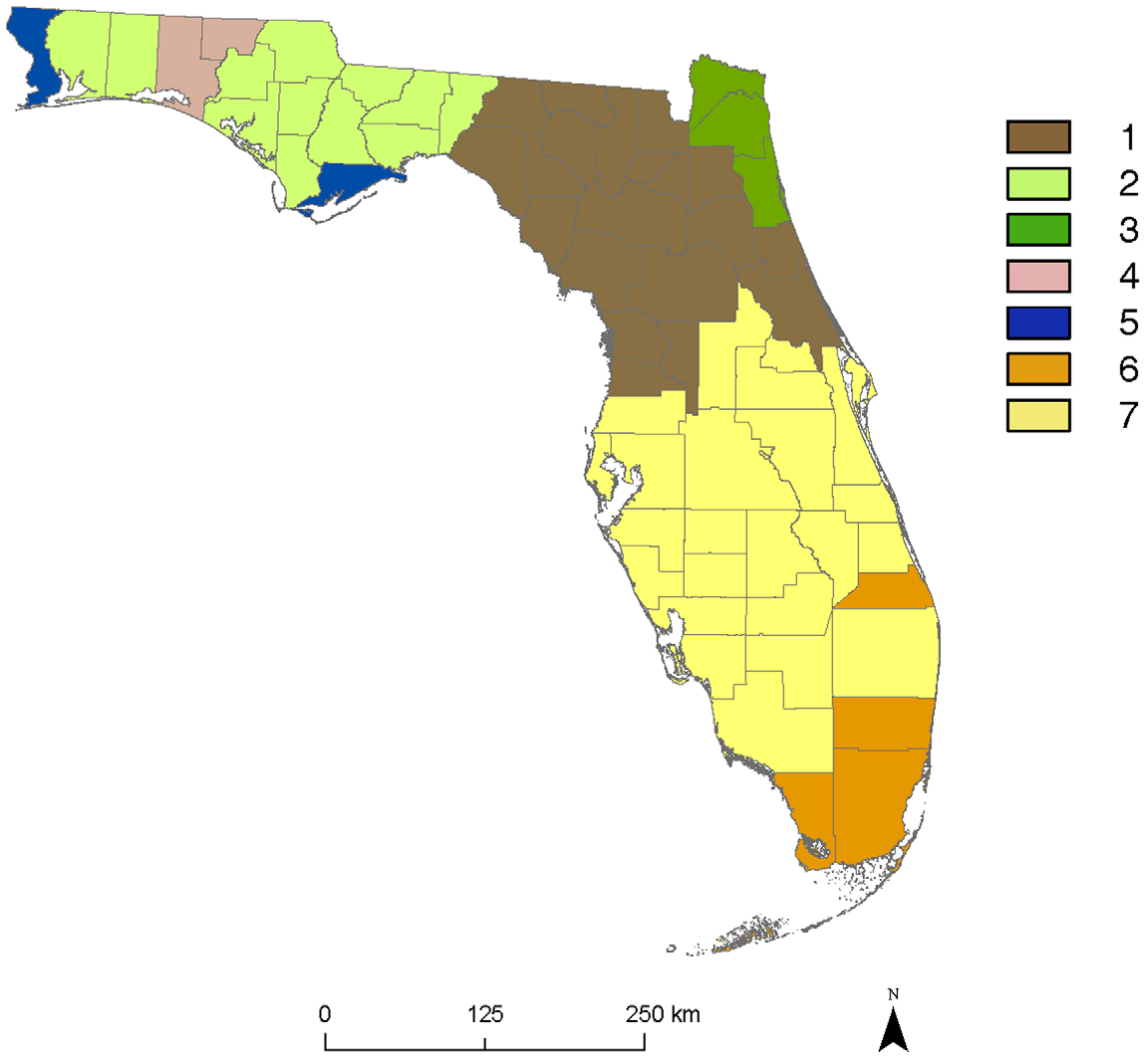
Florida counties grouped by similar climatic conditions. The climatic factors included monthly averages for minimum temperature, maximum temperature, and the precipitation for each county from 1973–2007. These seven clusters of counties had similar historic climatic trends that we treated as separate biogeographic regions for these analyses. See Table S3A for county identity in each biogeographic region.

To reduce spatial autocorrelation and climatic heterogeneity for the 70 species analyzed, we clustered the 67 Florida counties by similar historic climatic trends. We did this by performing a hierarchical cluster analysis, which groups counties based on a measure of similarity among county attributes, for the 57 counties with weather stations (see, e.g., [17]). To maximize the number of counties with climate data and to capture the period of greatest climatic change, we used records from 1973–2007 for the cluster analysis. We used the average monthly minimum temperature, maximum temperature, and precipitation data for each county (an average of 36 data points per year) from 1973–2007 as the attributes to group the most similar counties. We used average linkage clustering to compute the distance between clusters and Euclidian distance as the measure of similarity. We checked for variability of each factor and standardized the temperature and precipitation variables so that they had a mean of 0 and a variance of 1. We determined the optimal number of clusters for the 57 Florida counties by applying Mojena's stopping rule, which identifies the first stage in the dendrogram at which there is a large change in the distance between clusters [18]. We grouped the ten Florida counties without a weather station with the closest county with a weather station, as determined by the Florida Climate Center, Center for Ocean-Atmospheric Prediction Studies. This resulted in seven clusters of counties (Fig. 1, Table S3A) which had similar historic climatic trends that we treated as separate biogeographic regions for the following analyses.

#### Environmental drivers of flowering time

To understand if the flowering phenologies of nonnative and 41 closely-related native plant species have changed over time as well as the environmental drivers of these changes, we performed multiple regression analyses for all species within each biogeographic region using the Julian date of specimens that were in flower as the dependent variable. We matched flowering and county climate data for each specimen, using all available flowering and climatic data. If a plant flowered in a county without a weather station at that time, we did not use those data. Environmental cues for flowering time, such as temperature or precipitation, may occur several months prior to the conditions measured at the time of flowering [19]. To account for lagged flowering responses to environmental cues, we selected the lowest (min_T) and highest (max_T) average monthly temperatures in the months of the calendar year prior to flowering for each individual in the analysis. Thus, we selected the min_T and max_T for each specimen from January to the month of flowering of the same year. The independent variables used for the multiple regression model (hereafter described as “model 1”) were year, average precipitation for the month of flowering (precip), the lowest average monthly minimum temperature in the calendar year prior to flowering (min_T), the highest average monthly maximum temperature in the calendar year prior to flowering (max_T), range of minimum temperatures for the months previous to flowering of that calendar year (range_minT), range of maximum temperatures for the months previous to flowering of that calendar year (range_maxT), plant origin (native or nonnative), and two-way interactions (year*precip, year*min_T, year*max_T, precip*min_T, precip*maxT, precip*origin, year*origin, min_T*origin, max_T*origin, rangeminT*origin, range_maxT*origin) between these variables. The range in minimum temperatures were calculated in the following way: greatest ‘min_T’ minus the lowest ‘min_T’, which occurred from January to the flowering month of the year of flowering. The range for the maximum temperatures were calculated similarly, but using the ‘max’_T in place of the ‘min_T’.

For example, the native *Ardisia escallonioides* was found in Brevard county in 2006 and this specimen was in flower on June 18th, or Julian date 170. The average monthly minimum temperatures that occurred prior to this were 1 = 11.6, 2 = 10.2, 3 = 13.9, 4 = 18.3,5 = 19.2,6 = 22.3, with January = 1, February = 2,etc. Thus the lowest mean minimum temperature (min_T) selected for this specimen was 10.2, for February of 2006. The greatest mean minimum temperature in 2006 prior to this specimen flowering was 22.3, which occurred in June of that year. Thus, the range in minimum temperature for this species was (22.3−10.2 = 12.1). We adjusted for multiple significance tests by applying a sequential Bonferroni adjustment within each of the clusters [20].

Additionally, we evaluated a second model to explore other environmental cues on flowering, specifically the effect of temperature cues in the form of heat accumulation as well as the number of freezing days prior to flowering, hereafter described as “model 2”. We performed multiple regression analyses as described above, using the Julian date of specimens that were in flower as the dependent variable and the following independent variables: year, total precipitation the day of flowering (precip), the cumulative growing degree days in the calendar year prior to flowering (CGDD), the number of days below freezing (<0°C) in the calendar year prior to flowering (Freeze), plant origin and two-way interactions between these variables (Yr*Precip, Yr*CGDD, Yr*Freeze, Precip&CGDD, Precip*Freeze, CGDD*Freeze, Yr*Origin, CGDD*Origin, Freeze*Origin, Precip*Origin). To calculate growing degree days, or a measure of heat accumulation, we used the GDD equation described in Otto et al. (2007, [21]), with a base temperature of 10°C, and no ceiling temperature requirement. We then summed the GDD numbers between the first day of the year of flowering to the date that the specimen was flowering to calculate cumulative growing degree days (CGDD). Thus, for the *Ardisia escallonioides* example above, GDD numbers were summed from January 1, 2006 to the flowering date, June 18, 2006, to calculate the cumulative growing degree days (CGDD) for this specimen. Methods used to predict flowering responses to climatic conditions, such as cumulative growing degree days [21] and the range in minimum and maximum temperatures, rely on a greater number of data points as the calendar year progresses and may affect the results of the model.

We performed follow-up analyses to the multiple regression analyses described above with simple linear regressions between the flowering dates of all specimens in each region as well as flowering dates for each species within each region by the range in minimum temperatures, calculated for each specimen. Flowering data were log-transformed to account for non-linear responses of flowering date to the range in minimum temperatures as well as an increased range in the variation of flowering date with increasing minimum temperatures.

## Results

### Climatic trends by county

**Figure 2:**
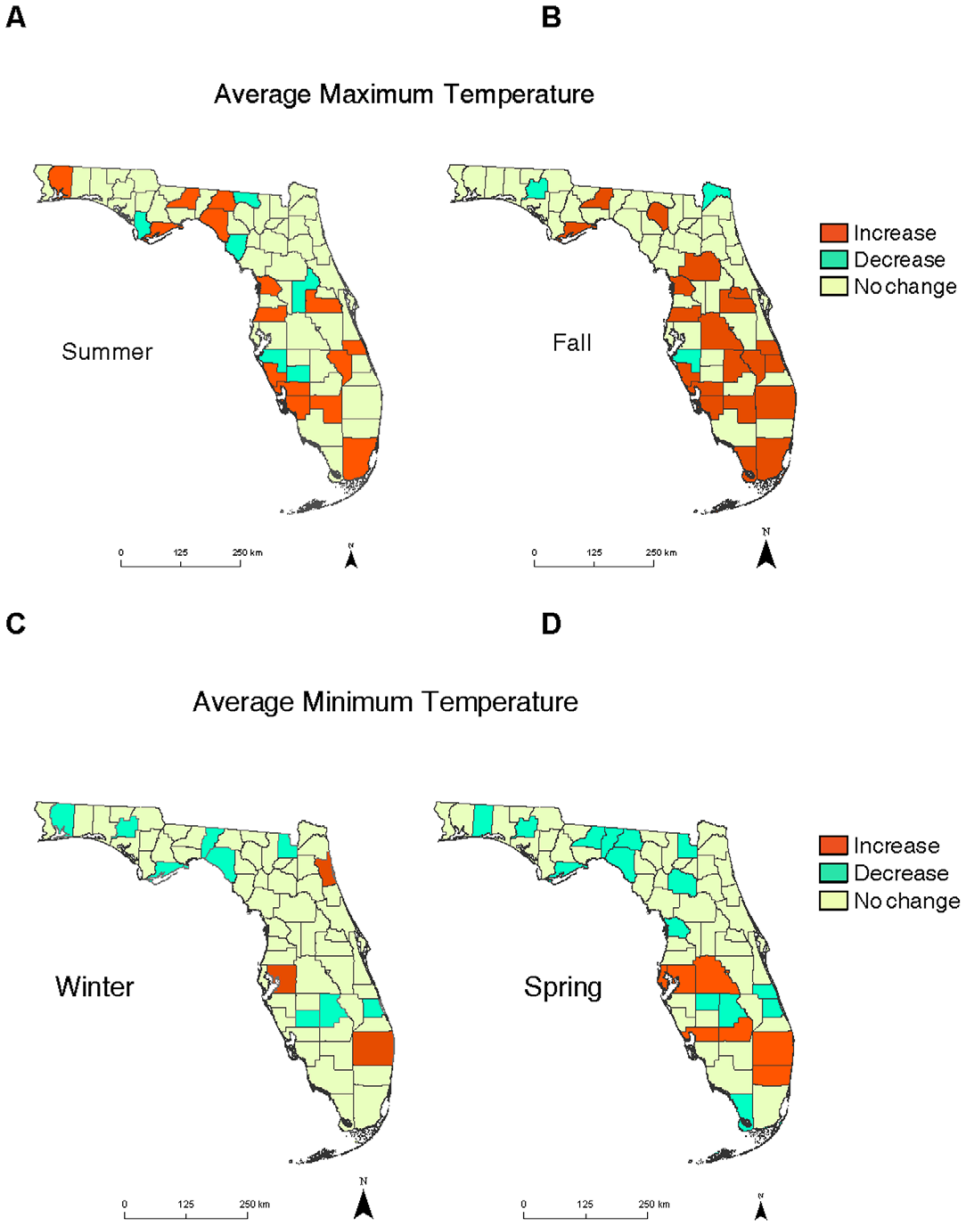
Historic temperature trends for Florida counties. Average maximum temperature trends for each Florida county in the (A) summer (June, July, August) and (B) fall (September, October, November) months. Average minimum temperature trends for each Florida county in the (C) winter (December, January, February) and (D) spring (March, April, May) months. Counties are colored for the change in average temperature over the monitored period, which ranged from 35 to 108 years. Orange indicates a significant increase, aqua denotes a significant decrease, and tan indicates no change in historical temperature for that county and season.

Historically, there has been a trend in Florida of warmer and wetter climate, with an average decadal temperature increase of 0.02°C (R^2^ = 0.025, p = 0.09) and precipitation increase of 0.75 cm (R^2^ = 0.022, p = 0.12) between 1895 and 2008 [2]. However, this overall warming trend for the state is complicated by warming and cooling trends that differ seasonally and by region. Over time Florida has been getting warmer in the summer and fall months, with 26% of counties experiencing significantly increased average maximum temperatures in the summer months (June, July, August) and 35% of the counties with significantly increased mean maximum temperatures in the fall months (September, October, November). These increases were within a range of 0.02 to 0.09°C per year for the last 35 to 108 years (Fig. 2, A and B, Table S4). The counties that experienced warmer fall maximum temperatures trends were clustered in southern Florida (Fig. 2B). Surprisingly, the majority of counties with significant changes in average monthly winter and spring temperatures had *decreased* minimum temperatures over the last century, with 16% of Florida counties recording significantly lower mean minimum temperatures in the winter months (December, January, February) and 26% of the counties documenting significantly lower average temperatures in the spring months (March, April, May). The counties with significantly lower winter and spring minimum temperatures tended to cluster in northern Florida (Fig. 2, C and D). The average monthly minimum temperature decreases ranged from -0.04 to −0.18°C per year for the last 34 to 108 years (Table S4). There were no significant differences in warming, cooling, or lack of trend by season for the weather stations with different durations of climatic data, suggesting that climatic trends did not differ by the length of time the weather station was in operation. Precipitation did not appear to change across most of Florida over time, with the majority of counties registering no significant seasonal changes in precipitation in the last 35 to 108 years (Table S5). However, significant increases in average monthly precipitation occurred in 11% of the counties in winter and summer months and significant decreases in monthly precipitation in 4% of the counties for the spring and summer months (Table S5).

### Patterns of flowering time

Across the state, two species flowered significantly later in the year (nonnative *Albizia lebbeck*, native *Sassafras albidum*) and one species flowered significantly earlier in the year (native *Morus rubra*) (Table S6).

### Biogeographic regions

In order to reduce spatial autocorrelation and climatic heterogeneity for the 70 species analyzed, we clustered the 67 Florida counties by similar historic climatic trends. This resulted in seven clusters of counties (Fig. 1, Table S3 A and B) which had similar historic climatic trends that we treated as separate biogeographic regions for the following analyses. In order to understand the effects of seasonal and regional climatic changes on plant reproduction in Florida, we analyzed flowering data for each of the Florida county clusters.

### Environmental drivers of flowering time

**Figure 3:**
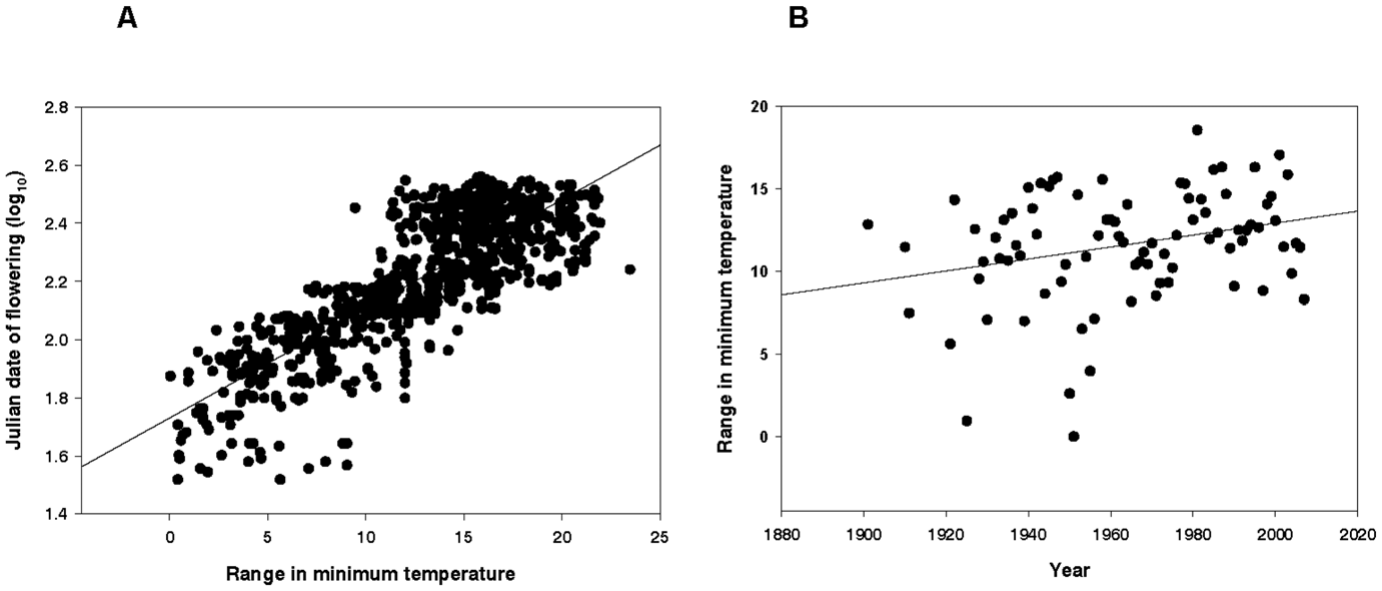
Phenological and climatic data, from region one. Region one was chosen as a typical representive of Florida regions, as all seven biogeographic regions displayed similar trends. A. Flowering date (log) increases linearly with the range in minimum temperature (p<0.0001, R^2^ = 0.69, n = 1,274). Each dot represents a single specimen, with the Julian date of flowering matched to the range in minimum temperature calculated for that specimen from climatic data from that county. B. The average range in minimum temperatures in region one increases over time (p = 0.009, R^2^ = 0.08, n = 86). Each dot represents a year of climatic data in region one, where the range in minimum temperatures in that region are matched with the year.

Plant flowering time was strongly delayed by variable minimum temperatures over historical time, with a range of approximately four to nineteen days later in the year, opposite the pattern observed for most phenological studies conducted worldwide [5], [22], [23]. Later flowering time was significantly correlated with the within-year variability in minimum temperatures, or the range of mean monthly minimum temperatures that occurred in the months of the calendar year prior to flowering, in all seven Florida biogeographic clusters (Fig. 3A, Table 1). Additionally, flowering time was delayed at the species level by greater variability in minimum temperatures (Table S7). Twenty-one of the seventy-nine simple linear regressions between flowering date and the range in minimum temperature for each species were statistically significant, after correcting for multiple comparisons. The regression coefficients for the range of minimum temperatures of these twenty-one significant regressions were positive, suggesting that as the range in minimum temperature increases in a given year, the flowering date for these species is significantly delayed (Table S7).Variability of minimum temperatures has increased in Florida over historical time (Fig. 3B).

**Table 1:** Analyses of the flowering time with climatic variables for each county cluster (model 1).

	Regression Parameters, by Cluster						
Response Variable	1	2	3	4	5	6	7
**Year**	1.17	2.04	−3.56	−1.56	1.81	1.63	2.76****
**Precip**	9.95	1.38	−209.70	46.94	−128.31	−14.32	−16.64
**min_T**	52.36	149.20	−1043.0	−251.34	−283.31	11.08	−66.16
**Max_T**	61.90	106.25	38.07	−106.22	133.94	156.55	238.26****
**Range_minT**	15.85****	4.07****	17.6***	13.49****	19.57***	12.38****	18.99****
**Range_maxT**	−3.39****	−1.17	−11.0	−5.37	0.73	1.57	−3.32*
**Origin**	414.63	−114.03	2767.06	12071.86	6524.23	18.26	−591.29
Yr*Precip	−0.004	0.001	0.11	−0.03	0.06	0.01	0.01
Yr*min_T	−0.02	−0.07	0.53	0.13	0.15	−0.002	0.04
Yr*max_T	−0.03	−0.05	−0.01	0.06	−0.07	−0.07	−0.12****
Precip*min_T	0.17*	−0.02	0.19	0.31	0.18	0.09	0.18****
Precip*max_T	−0.14*	−0.11	−0.08	0.21	0.18	−0.34****	−0.40****
Yr*Origin	−0.17	0.08	−1.31	−5.80	−3.42	−0.05	0.28
min_T*Origin	3.24	5.68	−12.10	16.24	−14.63	2.61	−1.72
max_T*Origin	−4.47	−4.89	−0.81	−40.71	17.83	2.08	2.45
Range_minT*Origin	6.69****	6.40	−6.87	46.06	−28.57	5.15	−0.09
Range_maxT*Origin	−3.31	1.37	4.50	0	12.95	−6.72	−1.06
Precip*Origin	−0.69	−1.48	−2.55	0	3.15	−1.05*	−0.05
**R^2^**	0.796	0.784	0.815	0.808	0.793	0.798	0.785
**p-value**	<.0001	<.0001	<.0001	<.0001	<.0001	<.0001	<.0001
**N**	1306	400	79	103	154	1055	1922

Response variables were year (yr), precipitation (precip), the lowest average monthly minimum temperature in the calendar year prior to flowering (min_T), the highest average monthly maximum temperature in the calendar year prior to flowering (max_T), range of minimum temperatures for the months previous to flowering of that calendar year (range_minT), range of maximum temperatures for the months previous to flowering of that calendar year (range_maxT), plant origin (native or nonnative; “origin”). Two-way interactions between these variables are listed by variable names separated by an ‘*’. Precipitation is in centimeters and temperature is in degrees Celsius. We applied a sequential Bonferroni adjustment within each cluster. *P<0.05, **P<0.001, *** *P*<0.001, **** *P*<0.0001, for tests of significant difference of parameter values from 0.

The two-way interaction of precipitation and minimum temperatures on flowering date was significant and positive in regions 1 and 7, suggesting that increased precipitation and average monthly minimum temperatures is correlated with plants flowering later in the year than expected from simply adding the effects of these two independent variables in these regions. In region 7, plant species flowered later in the year over historical time outside of the influence of the increased variability in minimum temperatures, as indicated by the statistical significance of the variable ‘year’. Thus, there may be an additional environmental factor that we did not measure that accounts for the later seasonal flowering in this region. The effect of the range of minimum temperatures differed by plant origin in region 1, with nonnative species flowering approximately 7 days later than native species. However, it should be noted that plant origin was not a significant factor explaining differences in flowering time in five of the seven biogeographic regions (2, 3, 4, 5, and 7) for model 1, suggesting that native and nonnative species did not differ in their flowering responses to these environmental factors in these regions during this time period.

In the statistical model (model 1), there were some environmental factors with negative correlation coefficients, implying that these factors would be associated with earlier flowering times (Table 1). The environmental factors responsible for trends to earlier flowering times were the interaction between average monthly precipitation levels and maximum temperatures (regions 1, 6, and 7), and the variability in maximum temperatures (regions 1 and 7). Additionally, the effect of precipitation differed by species origin in region 6, with nonnative plants flowering approximately one day earlier per 1 centimeter increase in precipitation than native plants. Last, the change in the variability of maximum temperatures over historical time was a significant factor that played a role for slightly earlier flowering times in regions 1 and 7. While these factors contributed to earlier flowering times, the primary driver of flowering time appears to be the range of minimum temperatures, whose effect estimate is much greater than range of maximum temperatures, and causes plants to flower later in the year. Among the variables considered, the range in minimum temperatures was the only environmental factor responsible for the change in plant flowering times in four regions (2, 3, 4, and 5), accounting for 78 to 81% of the variation in the flowering times of native and nonnative species. In sum, the environmental factor with the greatest correlation with plant flowering times in all seven regions was the variability in minimum temperatures, and this factor was strongly associated with later seasonal flowering times.

The second model, which included the effects of cumulative growing degree days and the number of freezes on flowering time, did not provide as clear of a signal for flowering time as the previous model which incorporated variability in minimum temperatures prior to flowering (Table S8). However the results of the second model supported the previous model (Table 1), which we highlight below. In regions two, four, and five, increasing numbers of freezing days prior to flowering delays flowering by plants to later in the year. In these three regions, this is tempered over time, as the effect of freeze dates on flowering declined over time, as indicated by the negative value of the interaction between the variables ‘Year’ and ‘Freeze’. Additionally, in region five, as the precipitation increases, this causes the effect of freeze dates to delay flowering even further, as indicated by the positive value of the interaction between the variables for precipitation and number of freezing days prior to flowering. The effect of cumulative growing degree days (CGDD) is greater for nonnative species in region six, as indicated by the positive term of the interaction between ‘CGDD’ and ‘Origin’.

To follow up on these results, we conducted simple linear regressions between the number of freezing days in each biogeographic region per calendar year over time. The number of freezing days have significantly increased over time in regions one, two, four, five, and seven [sample size, R^2^, regression coeffient (r.c.), and p-values presented by region: One, n = 964, R^2^ = 0.03, r.c. = 0.04, p = <0.0001; Two, n = 356, R^2^ = 0.07, r.c. = 0.13, p = <0.0001, Four, n = 75, R^2^ = 0.06, r.c. = 0.15, p = 0.04; Five, n = 138, R^2^ = 0.08, r.c. = 0.10, p = 0.0007, Seven, n = 1724, R^2^ = 0.005, r.c. = 0.009, p = 0.003], suggesting that the trend for increasing days below freezing per calendar year is common in Florida.

## Discussion

Contrary to expectation, the majority of counties with significant changes in average monthly winter and spring temperatures had *lower* minimum temperatures over historical time. Florida counties experienced significantly *colder* winter and springs while simultaneously experiencing significantly *warmer* summer fall temperatures over historical time. The warming trend in the fall was concentrated in southern counties while the cooling trends in the winter and spring were clustered in northern Florida. These results suggest that climate change varies by region and season. The strongest driver of these later flowering times in our study was the variability in the minimum temperatures, rather than simply the lowering of the minimum temperatures that occurred in 16% of the Florida counties in winter and 26% of the counties in spring months.

Only two species had significantly later flowering times (*Albizia lebbeck, Sassafras albidum*), and one species flowered significantly earlier in the season (*Morus rubra*) across the state. The low number of species that demonstrated significant changes in flowering time across the state in this analysis may owe to the different climatic conditions occurring in each individual biogeographic region. Each biogeographic region experienced distinct climatic conditions during the study period, and these environmental conditions had strong effects on plant reproduction. Thus, varying environmental influences of each biogeographic region on flowering time may be obscured when analyzed together across a large region.

Temperature is a limiting factor for reproduction, survival and growth in plants [24]. Low temperature extremes limit plants by frost or cold damage to leaves and buds or the freezing death of whole plants [15]. In this study, we found that greater variability in minimum temperatures was associated with strongly delayed flowering time in all seven biogeographic regions and that increasing freezing temperatures occurring prior to flowering were associated with later flowering times in three biogeographic regions. Thus, delays in flowering time associated with fluctuating minimum temperatures might have been caused by the lowest temperatures in the range, in other words, increased freezing temperatures might have been primarily responsible for delayed flowering. At the species level, both native and nonnative species experienced delayed flowering in years with large variability in minimum temperatures, suggesting that fluctuating minimum temperatures significantly delayed reproduction by plants. It may be difficult for plants to respond physiologically to large temperature fluctuations and so plants may cue their flowering times on the variation in minimum temperatures, rather than the lowest minimum temperatures, which could lead to bud dormancy extending later in the year. Whether plants cue their reproduction on extreme low temperatures or variability in low temperatures is an issue that needs to be explored further. The variation in minimum temperatures has increased over historical time in all regions of Florida. Additionally, the number of days occurring per calendar year below freezing has significantly increased over time in five of the seven biogeographic regions. If this trend continues, reproductive events by native and nonnative species may continue to be delayed to later in the season in Florida.

Worldwide, there are numerous examples where temperature changes have resulted in extended growing and reproductive periods which have provided nonnative species from warmer climates opportunities to expand and invade into new ranges [6]. In our study, the effect of the range of minimum temperatures differed by plant origin in region 1, with nonnative species flowering approximately 7 days later than native species. However, it should be noted that plant origin was not a significant factor explaining differences in flowering time in five of the seven biogeographic regions (2, 3, 4, 5, and 7) in model 1, implying that native and nonnative species did not differ in their flowering times in these regions during this time period. Likewise, in our second model, there were no differences between plant origins to freezing temperatures but there were differences in origin for heat accumulation, suggesting that nonnatives may be able to track warming temperatures more quickly than natives do [25]. This would suggest that nonnative plants in Florida do not have an overwhelmingly greater phenological response to climatic change in the form of increased freezing temperatures than closely related native species do, and may not be differentially favored by climate change in regions experiencing colder winter and spring conditions. However, nonnative species may be able to capitalize on warming conditions, as suggested by an empirical study of marine invertebrate species in the northeastern United States where nonnative recruitment was stronger than native species under conditions of warming spring water temperatures [26]. Likewise, non-native invasive species tracked seasonal temperature variation better than natives did in Massachusetts, flowering significantly earlier than natives with warming spring temperatures [25]. There are many examples from around the world where warming temperatures have resulted in extended growing and reproductive periods which have provided nonnative species from warmer climates opportunities to expand and invade into new ranges [6]. However, it is unknown what types of environmental conditions associated with climatic change that nonnatives will be able to capitalize on, as compared to native species [7]. Further research should be conducted to understand nonnative species responses to the specific environmental conditions (e.g. changes in temperature, precipitation, and nutrients) of climatic change in order to predict community composition under changing climatic conditions.

Climate change varies around the world and concomitant ecological responses are likely to differ by region [4]. However, the majority of research on global climate change and species phenology has been conducted in northern latitudes, ranging from 31.9 to 71.2° [27], with mean latitudes of 49.8 and 51.7 degrees reported by two of the most comprehensive meta-analyses of phenological responses of species to climate change ([5] and [12], respectively). A more recent meta-analysis of the phenological shifts due to climate change included 125,000 observational series of 542 plant and 19 animal species in 21 European countries[22] which ranged in latitude from 37.35° N to 69.75° [28]. In this study, 30% of the leafing, flowering, and fruiting records were significantly earlier while 3% were significantly delayed[22]. Phenological studies conducted in a temperate-subtropical climate are extremely rare. Florida's latitude ranges from 24° 30′ N to 31° N and it is possible that the delays in onset of species reproduction may be associated with environmental conditions of lower latitudes.

While the vast majority of spring events in mid- to high-latitudes have occurred earlier in the season and are associated with warming spring temperatures [22], delayed onset of spring phases have occurred in several cases. In the Balkans, leaf unfolding and flowering has been retarded in the time period from 1959 to 1993 [3]. In a phenological study of twelve plant and animal taxa in Japan and South Korea, first observations of a five of these species (frog, butterfly, wasp, and two bird species) were delayed over the time period of 1953–2005 at the majority of sites [29]. Interestingly, the sites from this study spanned a wide latitudinal gradient (24°20.2N to 45°24.9′N) including boreal as well as subtropical climates, rarely the focus of phenological studies.

Seasonal and regional differences in climatic changes strongly affect species reproductive phenologies and likely have cascading effects on the populations, communities and ecosystems of these regions [30]. While the greatest levels of warming of land and ocean surfaces are expected to occur in high latitudes[1], the complexity of air temperature changes in the subtropics found in this study warrants further attention. Furthermore, the velocity at which low-elevation regions with low topographic relief, such as Florida, will experience climate change is expected to be higher than in areas with greater topographic relief [31]. Species in regions with low topographic relief will require faster response times to climate change [31] and therefore these regions should be a high priority for research on species adaptations to climate change.

## Supporting Information

Table S1 The 29 high impact nonnative species and their 41 most closely related native species in Florida.
(0.05 MB DOC)

Table S2 The 57 Florida counties with weather stations, listed alphabetically.
(0.05 MB DOC)

Table S3 A. Florida counties clustered by similar climatic conditions, from 1973-2007. B. Average annual climatic variables for each biogeographic region, from 1973-2007.
(0.04 MB DOC)

Table S4 Historic trends in temperature by Florida county.
(0.03 MB DOC)

Table S5 Historic trends in average monthly precipitation levels by Florida county.
(0.03 MB DOC)

Table S6 Patterns in flowering time for nonnative and native species.
(0.07 MB DOC)

Table S7 Simple linear regression of the log of flowering date with range in minimum temperature for each species in each region.
(0.16 MB DOC)

Table S8 Analyses of the flowering time with cumulative growing degree days, freezing days, and precipitation climatic variables for each county cluster (model 2).
(0.05 MB DOC)
