# Supplementary material for: Climatic Variability Leads to Later Seasonal Flowering of Floridian Plants
Source: PLoS One. 2010 Jul 21;5(7):e11500. doi: 10.1371/journal.pone.0011500 (PMC2908116; doi:10.1371/journal.pone.0011500)
Supplement: Table S1 — The 29 high impact nonnative species and their 41 most closely related native species in Florida. (0.05 MB DOC) [file pone.0011500.s001.doc]

| **Plant family** | **Nonnative species** | **Native species** |
| --- | --- | --- |
| Fabaceae | *Abrus precatorius* | *Sesbania herbacea*  *Sesbania vesicaria* |
| Fabaceae | *Albizia julibrissin* | *Lysiloma latisliquum*  *Pithecellobium keyense* |
| Fabaceae | *Albizia lebbeck* | *Lysiloma latisliquum*  *Pithecellobium keyense* |
| Euphorbiaceae | *Aleurites fordii* | *Drypetes* *laterifolia* |
| Myrsinaceae | *Ardisia crenata* | *Ardisia escallonioides* |
| Aristolochiaceae | *Aristolochia littoralis* | *Aristolochia serpentaria*  *Aristolochia tomentosa* |
| Fabaceae | *Bauhnia variegata* | *Cercis canadensis* |
| Moraceae | *Broussonetia papyrifera* | *Morus rubra* |
| Casuarinaceae | *Casuarina cunninghamiana* | none in family |
| Casuarinaceae | *Casuarina equisitifolia* | none in family |
| Casuarinaceae | *Casuarina glauca* | none in family |
| Lauraceae | *Cinnamomum camphora* | *Sassafras albidum*  *Litsea aestivalis*  *Lindera benzoin* |
| Apocynaceae | *Cryptostegia madagascariensis* | *Cynanchum* *angustifolium*  *Cynanchum* *scoparium*  *Matelea* *floridana*  *Matelea* *gonocarpos*  *Matelea* *pubiflora* |
| Elaeagnaceae | *Eleagnus pungens* | none in family |
| Acanthaceae | *Hygrophila polysperma* | *Hygrophila costata* |
| Poaceae | *Hymenanche amplexicaulis* | *Stenotaphrum secundatum*  *Reimarochloa oligostachya*  *Digitaria cognata*  *Digitaria filiformis var. filiformis*  *Digitaria insularis*  *Digitaria serotina* |
| Oleaceae | *Jasminium fluminense* | *Osmanthus americanus* |
| Sapindaceae | *Koelreuteria elegans* | *Acer* *negundo*  *Acer rubrum*  *Acer* *saccharum subsp. floridanum*  *Exothea* *paniculata* |
| Table S1, *Continued* | | |
| **Plant family** | **Nonnative species** | **Native species** |
| Oleaceae | *Ligustrum sinense* | *Osmanthus americanus* |
| Arecaceae | *Livistona chinensis* | *Serenoa* *repens* |
| Bignoniaceae | *Macfadyena unguis-cati* | *Bignonia capreolata* |
| Meliaceae | *Melia azeradach* | none in family |
| Berberidaceae | *Nandina domestica* | *Podophyllum peltatum* |
| Arecaceae | *Phoenix reclinata* | *Serenoa repens*  *Sabal minor*  *Sabal palmetto*  *Rhapidophyllum hystrix* |
| Myrtaceae | *Rhodomyrtus tomentosus* | *Calyptranthes pallens* |
| Ruscaceae | *Sanservieria hyacinthoides* | *Polygonatum biflorum* |
| Euphorbiaceae | *Sapium sebiferum* | *Sebastiana fruticosa* |
| Arecaceae | *Syagrus romanzoffiana* | *Serenoa repens*  *Sabal minor*  *Sabal palmetto*  *Rhapidophyllum hystrix* |
| Lamiaceae | *Vitex trifolia* | *Callicarpa americana*  *Conradina canescens* |
