# Supplementary material for: Climatic Variability Leads to Later Seasonal Flowering of Floridian Plants
Source: PLoS One. 2010 Jul 21;5(7):e11500. doi: 10.1371/journal.pone.0011500 (PMC2908116; doi:10.1371/journal.pone.0011500)
Supplement: Table S2 — The 57 Florida counties with weather stations, listed alphabetically. (0.05 MB DOC) [file pone.0011500.s002.doc]

| **County** | **years** | **County** | **years** |
| --- | --- | --- | --- |
| Alachua | 64 | Leon | 60 |
| Baker | 89 | Levy | 52 |
| Bay | 36 | Madison | 105 |
| Brevard | 107 | Manatee | 51 |
| Broward | 96 | Marion | 108 |
| Charlotte | 94 | Martin | 72 |
| Citrus | 90 | Monroe | 72 |
| Collier | 83 | Nassau | 107 |
| Columbia | 108 | Okaloosa | 71 |
| Dade | 73 | Okeechobee | 70 |
| DeSoto | 108 | Orange | 53 |
| Dixie | 60 | Osceola | 49 |
| Duval | 64 | Palm Beach | 108 |
| Escambia | 60 | Pasco | 108 |
| Franklin | 77 | Pinellas | 108 |
| Gadsden | 40 | Polk | 108 |
| Glades | 89 | Putnam | 108 |
| Gulf | 57 | Santa Rosa | 60 |
| Hamilton | 58 | Sarasota | 55 |
| Hardee | 75 | Seminole | 60 |
| Hendry | 79 | St. Johns | 35 |
| Hernando | 108 | St. Lucie | 107 |
| Highlands | 107 | Sumter | 68 |
| Hillsborough | 105 | Suwannee | 73 |
| Indian River | 66 | Taylor | 72 |
| Jefferson | 103 | Volusia | 108 |
| Lafayette | 59 | Walton | 108 |
| Lake | 60 | Washington | 69 |
| Lee | 103 |  |  |

The number of years of operation of weather stations is listed to the right of the county name.
