# Supplementary material for: Climatic Variability Leads to Later Seasonal Flowering of Floridian Plants
Source: PLoS One. 2010 Jul 21;5(7):e11500. doi: 10.1371/journal.pone.0011500 (PMC2908116; doi:10.1371/journal.pone.0011500)
Supplement: Table S3 — A. Florida counties clustered by similar climatic conditions, from 1973-2007. B. Average annual climatic variables for each biogeographic region, from 1973-2007. (0.04 MB DOC) [file pone.0011500.s003.doc]

A.

| **Biogeographic region** | **Counties in each biogeographic region** |
| --- | --- |
| **1** | Alachua, Bradford, Levy, Lafayette, Suwannee, Taylor, Madison, Gilchrist, Baker, Hamilton, Columbia, Sumter, Dixie, Citrus, Hernando, Putnam, Marion, Volusia, Clay, Flagler, Union |
| **2** | Calhoun, Gadsden, Liberty, Leon, Gulf, Bay , Jackson, Jefferson, Okaloosa, Santarosa, Wakulla, Washington |
| **3** | Duval, Nassau, St. Johns |
| **4** | Holmes, Walton |
| **5** | Escambia, Franklin |
| **6** | Broward, Dade, Miami-Dade, Monroe, Martin |
| **7** | Brevard, Glades, Okeechobee, Palm Beach, Indian River, St. Lucie, De Soto, Highlands, Hardee, Hendry, Hillsoborough, Manatee, Polk, Pasco, Osceola, Lake, Seminole, Orange, Charlotte, Sarasota, Collier, Lee, Pinellas |

B.

| **Biogeographic region** | **Min. T (°C)** | **Max. T (°C)** | **Range Min. T** | **Range Max. T** | **Precip. (cm)** |
| --- | --- | --- | --- | --- | --- |
| **1** | 5.5 | 30.9 | 13.0 | 11.2 | 11.0 |
| **2** | 2.9 | 28.2 | 12.7 | 11.7 | 12.6 |
| **3** | 7.1 | 30.5 | 13.3 | 11.9 | 11.1 |
| **4** | 2.7 | 28.7 | 12.0 | 11.9 | 8.6 |
| **5** | 5.2 | 29.4 | 15.0 | 13.5 | 13.9 |
| **6** | 13.9 | 30.1 | 7.6 | 5.9 | 10.5 |
| **7** | 8.5 | 30.5 | 10.4 | 9.0 | 10.5 |
|  |  |  |  |  |  |

Minimum temperature, maximum temperatures, range in minimum temperatures, range in maximum temperatures, and precipitation are presented.

See Figure 1 for corresponding location of biogeographic region.
