# Supplementary material for: Climatic Variability Leads to Later Seasonal Flowering of Floridian Plants
Source: PLoS One. 2010 Jul 21;5(7):e11500. doi: 10.1371/journal.pone.0011500 (PMC2908116; doi:10.1371/journal.pone.0011500)
Supplement: Table S4 — Historic trends in temperature by Florida county. (0.03 MB DOC) [file pone.0011500.s004.doc]

| **Season** | **Climatic factor (means)** | **No. counties increase (%)** | **Range of increase/yr (°C)** | **No. counties decrease (%)** | **Range of decrease/yr (°C)** | **No. counties no change(%)** | **Range of years obs.** |
| --- | --- | --- | --- | --- | --- | --- | --- |
| Winter | Minimum Temperature | 3 (5) | 0.05 - 0.18 | 9 (16) | 0.04 - 0.18 | 45 (79) | 34-108 |
| Spring | Minimum Temperature | 7 (12) | 0.04 - 0.11 | 15 (26) | 0.04 - 0.11 | 36 (63) | 35-108 |
| Summer | Maximum Temperature | 15 (26) | 0.02 – 0.09 | 6 (11) | 0.02 – 0.07 | 36 (63) | 35-108 |
| Fall | Maximum Temperature | 20 (35) | 0.02 – 0.09 | 3 (5) | 0.05 – 0.05 | 35 (61) | 38-108 |

The number of counties with statistically significant increases (# counties increase), significant decreases (# counties decrease), and no significant changes (# counties no change) are listed. The percentages of those counties out of the total 57 Florida counties with climate data which experienced significant increases, decreases and no changes are in parenthesis.
