# Supplementary material for: Climatic Variability Leads to Later Seasonal Flowering of Floridian Plants
Source: PLoS One. 2010 Jul 21;5(7):e11500. doi: 10.1371/journal.pone.0011500 (PMC2908116; doi:10.1371/journal.pone.0011500)
Supplement: Table S6 — Patterns in flowering time for nonnative and native species. (0.07 MB DOC) [file pone.0011500.s006.doc]

| **Species** | **Origin** | **# Non-flwring** | **#Flwring** | **Earliest**  **record** | **Latest record** | **Diff. in avg. flwr. time** |
| --- | --- | --- | --- | --- | --- | --- |
| *Albizia lebbeck* | E | 32 | 25 | 1931 | 2006 | 46* |
| *Bauhnia variegata* | E | 13 | 41 | 1928 | 2005 | -5 |
| *Broussonetia papyrifera* | E | 21 | 53 | 1932 | 2005 | 24 |
| *Casuarina equisetifolia* | E | 81 | 87 | 1895 | 2005 | 8 |
| *Jasminium fluminense* | E | 7 | 26 | 1937 | 2004 | -32 |
| *Ligustrum sinense* | E | 23 | 35 | 1933 | 2006 | -3 |
| *Melia azedarach* | E | 83 | 61 | 1893 | 2007 | 2 |
| *Rhodomyrtus tomentosus* | E | 24 | 39 | 1893 | 2005 | 2 |
| *Sansevieria hyacinthoides* | E | 16 | 31 | 1903 | 2005 | -32 |
| *Vitex trifolia* | E | 17 | 56 | 1940 | 2007 | 42 |
| *Acer rubrum* | N | 309 | 54 | 1892 | 2008 | 18 |
| *Ardisia escallonioides* | N | 140 | 71 | 1895 | 2006 | -9 |
| *Bignonia capreolata* | N | 34 | 62 | 1876 | 2007 | 3 |
| *Callicarpa americana* | N | 228 | 152 | 1893 | 2007 | -8 |
| *Cercis canadensis* | N | 41 | 38 | 1928 | 2008 | -0.3 |
| *Conradina canescens* | N | 30 | 112 | 1920 | 2002 | -12 |
| *Cynanchum angustifolium* | N | 13 | 128 | 1894 | 2006 | 7 |
| *Cynanchum scoparium* | N | 40 | 82 | 1895 | 2005 | -17 |
| *Exothea paniculata* | N | 55 | 36 | 1897 | 2006 | 1 |
| *Lysiloma latisliquum* | N | 38 | 34 | 1913 | 2001 | -9 |
| *Matelea* *floridana* | N | 4 | 32 | 1897 | 2007 | 8 |
| *Matelea* *pubiflora* | N | 11 | 23 | 1897 | 1995 | 17 |
| *Morus rubra* | N | 124 | 42 | 1893 | 2006 | -28* |
| *Osmanthus americanus* | N | 170 | 117 | 1896 | 2007 | 6 |
| *Pithecellobium keyense* | N | 55 | 47 | 1913 | 2005 | -22 |
| *Polygonatum biflorum* | N | 20 | 21 | 1928 | 2002 | 10 |
| *Sabal minor* | N | 99 | 43 | 1896 | 2008 | -33 |
| *Sabal palmetto* | N | 133 | 71 | 1918 | 2006 | -10 |
| *Sassafras albidum* | N | 61 | 29 | 1929 | 2007 | 9* |
| *Serenoa repens* | N | 228 | 158 | 1894 | 2007 | 10 |
| *Sesbania herbacea* | N | 44 | 81 | 1918 | 2007 | -9 |
| *Sesbania vesicaria* | N | 53 | 94 | 1894 | 2007 | 10 |
| *Stenotaphrum secundatum* | N | 45 | 62 | 1876 | 2005 | -23 |

Patterns of flowering time for the most abundant native and nonnative species, or those which had at least 10 specimens available for each the earlier and later time period. Species origin is listed by (E) for nonnative, (N) for native, number of nonflowering specimens available for each species, number of specimens in flower, the earliest record, and the latest record for each species. The sign of the difference in the average flowering time (Average flowering time from 1890-1969 minus average flowering time from 1970-2007) is reversed so that positive values indicate delays in flowering time, and negative values indicate earlier seasonal flowering times.
