# Supplementary material for: Climatic Variability Leads to Later Seasonal Flowering of Floridian Plants
Source: PLoS One. 2010 Jul 21;5(7):e11500. doi: 10.1371/journal.pone.0011500 (PMC2908116; doi:10.1371/journal.pone.0011500)
Supplement: Table S7 — Simple linear regression of the log of flowering date with range in minimum temperature for each species in each region. (0.16 MB DOC) [file pone.0011500.s007.doc]

| **Species** | **Origin** | **Region** | **n** | **R^2^** | **Regression coefficient** | **p-value** |
| --- | --- | --- | --- | --- | --- | --- |
| *Abrus precatorius* | E | 6 | 26 | 0.10 | 0.06 | 0.12 |
| ***Abrus precatorius*** | **E** | **7** | **67** | **0.27** | **0.09** | **<0.0001** |
| *Albizia julibrissin* | E | 1 | 24 | 0.04 | -0.01 | 0.38 |
| *Albizia lebbeck* | E | 6 | 24 | 0.29 | 0.10 | 0.006 |
| ***Albizia lebbeck*** | **E** | **7** | **21** | **0.67** | **0.22** | **<0.0001** |
| *Ardisia crenata* | E | 1 | 26 | 0.11 | 0.06 | 0.09 |
| *Ardisia crenata* | E | 7 | 22 | 0.25 | 0.08 | 0.02 |
| *Ardisia escallonioides* | E | 6 | 64 | 0.16 | 0.07 | 0.001 |
| *Ardisia escallonioides* | E | 7 | 105 | 0.05 | 0.05 | 0.02 |
| *Aristolochia littoralis* | E | 7 | 16 | 0.11 | 0.07 | 0.22 |
| *Bauhnia variegata* | E | 6 | 25 | 0.14 | 0.07 | 0.06 |
| *Bauhnia variegata* | E | 7 | 17 | 0.24 | 0.05 | 0.05 |
| *Broussonetia papyrifera* | E | 1 | 18 | 0.43 | 0.05 | 0.003 |
| *Broussonetia papyrifera* | E | 7 | 16 | 0.04 | 0.02 | 0.46 |
| *Casuarina cunninghamiana* | E | 6 | 15 | 0.14 | 0.11 | 0.18 |
| *Casuarina cunninghamiana* | E | 7 | 15 | 0.06 | 0.03 | 0.39 |
| *Casuarina equisetifolia* | E | 6 | 66 | 0.15 | 0.10 | 0.001 |
| ***Casuarina equisetifolia*** | **E** | **7** | **56** | **0.33** | **0.11** | **<0.0001** |
| *Casuarina glauca* | E | 6 | 22 | 0.31 | 0.13 | 0.007 |
| *Casuarina glauca* | E | 7 | 25 | 0.01 | 0.01 | 0.61 |
| *Cinnamomum camphora* | E | 1 | 27 | 0.07 | 0.03 | 0.19 |
| *Cinnamomum camphora* | E | 7 | 37 | 0.06 | 0.04 | 0.14 |
| *Eleagnus pungens* | E | 2 | 19 | 0.16 | 0.07 | 0.09 |
| *Hygrophila polysperma* | E | 7 | 16 | 0.02 | 0.02 | 0.65 |
| *Hymenanche amplexicaulis* | E | 7 | 19 | 0.004 | 0.01 | 0.81 |
| *Jasminium fluminense* | E | 6 | 19 | 0.25 | 0.08 | 0.03 |
| *Ligustrum sinense* | E | 2 | 15 | 0.18 | -0.03 | 0.11 |
| *Melia azedarach* | E | 1 | 31 | 0.15 | 0.05 | 0.04 |
| *Melia azedarach* | E | 6 | 15 | 0.07 | 0.05 | 0.33 |
| ***Melia azedarach*** | **E** | **7** | **42** | **0.37** | **0.09** | **<0.0001** |
| ***Rhodomyrtus tomentosus*** | **E** | **7** | **38** | **0.43** | **0.07** | **<0.0001** |
| *Sansevieria hyacinthoides* | E | 7 | 24 | 0.27 | 0.12 | 0.01 |
| *Sapium sebiferum* | E | 1 | 19 | 0.03 | 0.02 | 0.47 |
| ***Vitex trifolia*** | **E** | **6** | **21** | **0.46** | **0.17** | **0.0007** |
| *Vitex trifolia* | E | 7 | 33 | 0.17 | 0.07 | 0.02 |
| *Acer negundo* | N | 1 | 30 | 0.03 | 0.02 | 0.36 |
| ***Acer rubrum*** | **N** | **1** | **56** | **0.55** | **0.08** | **<0.0001** |
| *Acer rubrum* | N | 2 | 43 | 0.02 | 0.02 | 0.32 |
| ***Acer rubrum*** | **N** | **7** | **114** | **0.31** | **0.08** | **<0.0001** |
| *Aristolochia serpentaria* | N | 1 | 21 | 0.06 | 0.04 | 0.28 |
| *Bignonia capreolata* | N | 1 | 25 | 0.21 | 0.04 | 0.02 |
| *Callicarpa americana* | N | 1 | 73 | 0.08 | -0.02 | 0.02 |
| Table S7, part (2) of 3 | | | | | | |
| **Species** | **Origin** | **Region** | **n** | **R^2^** | **Regression coefficient** | **p-value** |
| *Callicarpa americana* | N | 2 | 19 | 0.01 | 0.01 | 0.62 |
| *Callicarpa americana* | N | 6 | 46 | 0.17 | 0.07 | 0.004 |
| *Callicarpa americana* | N | 7 | 129 | 0.006 | 0.007 | 0.41 |
| *Calyptranthes pallens* | N | 6 | 27 | 0.04 | 0.06 | 0.30 |
| ***Cercis canadensis*** | **N** | **1** | **31** | **0.43** | **0.06** | **<0.0001** |
| ***Conradina canescens*** | **N** | **2** | **30** | **0.39** | **0.05** | **0.0002** |
| *Conradina canescens* | N | 5 | 25 | 0.01 | 0.01 | 0.64 |
| *Conradina canescens* | N | 7 | 36 | 0.09 | 0.03 | 0.07 |
| ***Cynanchum angustifolium*** | **N** | **1** | **20** | **0.72** | **0.07** | **<0.0001** |
| *Cynanchum angustifolium* | N | 6 | 28 | 0.16 | 0.08 | 0.04 |
| ***Cynanchum angustifolium*** | **N** | **7** | **42** | **0.53** | **0.08** | **<0.0001** |
| *Cynanchum scoparium* | N | 1 | 21 | 0.07 | 0.02 | 0.2431 |
| *Cynanchum scoparium* | N | 6 | 17 | 0.03 | 0.03 | 0.50 |
| ***Cynanchum scoparium*** | **N** | **7** | **52** | **0.23** | **0.09** | **0.0003** |
| *Digitaria filiformis var. filiformis* | N | 1 | 16 | 0.10 | -0.01 | 0.24 |
| *Digitaria filiformis var. filiformis* | N | 7 | 34 | 0.05 | 0.03 | 0.21 |
| *Digitaria insularis* | N | 6 | 17 | 0.49 | 0.16 | 0.0017 |
| ***Digitaria serotina*** | **N** | **7** | **23** | **0.72** | **0.08** | **<0.0001** |
| ***Drypetes* *laterifolia*** | **N** | **6** | **26** | **0.41** | **0.11** | **0.0004** |
| ***Exothea paniculata*** | **N** | **6** | **46** | **0.33** | **0.06** | **<0.0001** |
| *Lysiloma latisliquum* | N | 6 | 52 | 0.16 | 0.05 | 0.003 |
| *Matelea* *floridana* | N | 1 | 15 | 0.27 | 0.04 | 0.05 |
| ***Morus rubra*** | **N** | **1** | **29** | **0.43** | **0.06** | **0.0001** |
| *Morus rubra* | N | 6 | 20 | 0.22 | 0.07 | 0.04 |
| ***Morus rubra*** | **N** | **7** | **59** | **0.34** | **0.06** | **<0.0001** |
| ***Osmanthus americanus*** | **N** | **1** | **58** | **0.29** | **0.06** | **<0.0001** |
| *Osmanthus americanus* | N | 2 | 28 | 0.27 | 0.04 | 0.005 |
| *Osmanthus americanus* | N | 5 | 15 | 0.08 | 0.03 | 0.32 |
| ***Osmanthus americanus*** | **N** | **7** | **48** | **0.26** | **0.06** | **0.0002** |
| *Pithecellobium keyense* | N | 6 | 68 | 0.06 | 0.06 | 0.04 |
| *Rhapidophyllum hystrix* | N | 1 | 28 | 0.02 | 0.03 | 0.43 |
| ***Rhapidophyllum hystrix*** | **N** | **7** | **52** | **0.25** | **0.10** | **0.0001** |
| *Sabal minor* | N | 1 | 42 | 0.11 | 0.02 | 0.03 |
| *Sabal minor* | N | 7 | 28 | 0.25 | 0.08 | 0.006 |
| *Sabal palmetto* | N | 1 | 30 | 0.18 | 0.04 | 0.02 |
| *Sabal palmetto* | N | 6 | 20 | 0.08 | 0.04 | 0.23 |
| *Sabal palmetto* | N | 7 | 48 | 0.02 | 0.02 | 0.30 |
| *Sassafras albidum* | N | 2 | 21 | 0.03 | 0.01 | 0.47 |
| *Sebastiana fruticosa* | N | 1 | 19 | 0.38 | 0.05 | 0.005 |
| *Serenoa repens* | N | 1 | 51 | 0.10 | 0.03 | 0.03 |
| *Serenoa repens* | N | 6 | 34 | 0.13 | 0.07 | 0.04 |
| Table S7, part (3) of 3 | | | | | | |
| **Species** | **Origin** | **Region** | **n** | **R^2^** | **Regression coefficient** | **p-value** |
| *Serenoa repens* | N | 7 | 133 | 0.06 | 0.04 | 0.003 |
| *Sesbania herbacea* | N | 1 | 18 | 0.009 | 0.005 | 0.72 |
| *Sesbania herbacea* | N | 6 | 23 | 0.31 | 0.09 | 0.005 |
| *Sesbania herbacea* | N | 7 | 52 | 0.04 | 0.03 | 0.17 |
| *Sesbania vesicaria* | N | 1 | 34 | 0.17 | -0.02 | 0.02 |
| *Sesbania vesicaria* | N | 7 | 55 | 0.07 | -0.2 | 0.05 |
| *Stenotaphrum secundatum* | N | 6 | 22 | 0.19 | 0.06 | 0.04 |
| *Stenotaphrum secundatum* | N | 7 | 39 | 0.08 | 0.04 | 0.09 |
|  |  |  |  |  |  |  |

Linear regressions of the log of flowering dates by the range in minimum temperature for each species in each biogeographic region. Only those species for which there were more than fifteen records per region are presented. Origin denotes nonnative (E) or native (N). Region denotes the biogeographic region in Florida (see Figure 2). The regression coefficient provided is that for relationship between the flowering date and the range of minimum temperatures. The p-value provided is for the model. Ninety-one regressions were run for an initial table wide alpha value of 0.00055, values in bold are those that were significant after a sequential Bonferroni correction.
