# Supplementary material for: Climatic Variability Leads to Later Seasonal Flowering of Floridian Plants
Source: PLoS One. 2010 Jul 21;5(7):e11500. doi: 10.1371/journal.pone.0011500 (PMC2908116; doi:10.1371/journal.pone.0011500)
Supplement: Table S8 — Analyses of the flowering time with cumulative growing degree days, freezing days, and precipitation climatic variables for each county cluster (model 2). (0.05 MB DOC) [file pone.0011500.s008.doc]

|  | | **Regression Parameters, by Cluster** | | | | | | |
| --- | --- | --- | --- | --- | --- | --- | --- | --- |
| **Response Variable** | 1 | | 2 | 3 | 4 | 5 | 6 | 7 |
| **Year** | -0.04 | | 0.29 | 0.38 | 0.47 | 0.28 | 0.01 | 0.08 |
| **Precip** | -22.16 | | 112.03 | 257.03 | -495.09 | 1044.35 | 20.63 | 23.86 |
| **CGDD** | 0.03 | | -0.07 | 0.60 | -0.44 | -0.21 | 0.18**** | 0.16**** |
| **Freeze** | 23.52 | | 41.98** | -46.30 | 87.38** | 173.25** | 303.94 | 45.23 |
| **Origin** | 307.30 | | 707.02** | -487.17 | -12515.43 | -2546.13** | 136.84 | 86.07 |
| **Yr*Precip** | 0.01 | | -0.06 | -0.13 | 0.25 | -0.55 | -0.008 | -0.01 |
| **Yr*CGDD** | 0.000 | | 0.0001 | -0.0003 | 0.0002 | 0.0001 | -0.0001*** | -0.0001*** |
| **Yr*Freeze** | -0.01 | | -0.02** | 0.03 | -0.04** | -0.09*** | -0.15 | -0.02 |
| **Precip* CGDD** | -0.002** | | -0.0003 | -0.003 | 0.007 | 0.01*** | -0.002**** | 0.0001 |
| **Precip*Freeze** | 0.06 | | 0.12 | 0.49 | -0.004 | 3.85*** | 0.42 | -0.06 |
| **CGDD*Freeze** | -0.0001 | | -0.0001 | -0.001 | -0.001 | 0.0001 | -0.0003 | -0.0002 |
| **Yr*Origin** | -0.16 | | -0.36** | 0.26 | 6.26 | 1.28** | -0.07 | -0.04 |
| **CGDD*Origin** | 0.0001 | | -0.001 | -0.01 | 0.04 | 0.001 | 0.002*** | 0.0006 |
| **Freeze*Origin** | 0.14 | | 0.52 | 0.71 | 4.06 | 0.83 | -1.09 | 0.54 |
| **Rain*Origin** | -0.75 | | -1.64 | -15.49 | -86.20 | -100.08 | 0.02 | 0.34 |
|  |  | |  |  |  |  |  |  |
| **R^2^** | 0.961 | | 0.976 | 0.982 | 0.976 | 0.981 | 0.986 | 0.979 |
| **p-value** | <.0001 | | <.0001 | <.0001 | <.0001 | <.0001 | <.0001 | <.0001 |
| **n** | 965 | | 356 | 41 | 75 | 138 | 895 | 1714 |

Response variables were year (yr), precipitation (precip), the cumulative growing degree days in the calendar year prior to flowering (CGDD), the number of days below freezing in the calendar year prior to flowering (Freeze), plant origin (native or nonnative; “origin”). Two-way interactions between these variables are listed by variable names separated by an ‘*’. Precipitation is in centimeters and temperature is in degrees Celsius. We applied a sequential Bonferroni adjustment within each cluster. **P<0.01, *** *P* <0.001, **** *P* <0.0001, for tests of significant difference of parameter values from 0.
